# Design of Surface Modifications for Nanoscale Sensor Applications

**DOI:** 10.3390/s150101635

**Published:** 2015-01-14

**Authors:** Erik Reimhult, Fredrik Höök

**Affiliations:** 1 Institute for Biologically Inspired Materials, Department of Nanobiotechnology, University of Natural Resources and Life Sciences, Vienna, Muthgasse 11, A-1190 Vienna, Austria; 2 Biological Physics, Department of Applied Physics, Chalmers University of Technology, Fysikgränd 3, SE-411 33 Göteborg, Sweden; E-Mail: fredrik.hook@chalmers.se

**Keywords:** nanoscale sensor, biosensor, surface functionalization, molecular patterning

## Abstract

Nanoscale biosensors provide the possibility to miniaturize optic, acoustic and electric sensors to the dimensions of biomolecules. This enables approaching single-molecule detection and new sensing modalities that probe molecular conformation. Nanoscale sensors are predominantly surface-based and label-free to exploit inherent advantages of physical phenomena allowing high sensitivity without distortive labeling. There are three main criteria to be optimized in the design of surface-based and label-free biosensors: (i) the biomolecules of interest must bind with high affinity and selectively to the sensitive area; (ii) the biomolecules must be efficiently transported from the bulk solution to the sensor; and (iii) the transducer concept must be sufficiently sensitive to detect low coverage of captured biomolecules within reasonable time scales. The majority of literature on nanoscale biosensors deals with the third criterion while implicitly assuming that solutions developed for macroscale biosensors to the first two, equally important, criteria are applicable also to nanoscale sensors. We focus on providing an introduction to and perspectives on the advanced concepts for surface functionalization of biosensors with nanosized sensor elements that have been developed over the past decades (criterion (iii)). We review in detail how patterning of molecular films designed to control interactions of biomolecules with nanoscale biosensor surfaces creates new possibilities as well as new challenges.

## 1. Introduction

One of the major remaining challenges in the development of bioanalytical sensors is to improve the efficiency by which low molecular weight biomolecules can be detected at utterly low concentrations in a complex biological fluid. Considerable efforts have been put into increasing the sensitivity of the transducer principles utilized in different biosensing devices. The sensor signal is generally proportional to surface coverage, which typically is directly dependent on the affinity of the interaction and bulk concentration of target molecules; the lower the affinity or concentration, the lower becomes the surface coverage. It is therefore critical that the sensor concept is sufficiently sensitive to detect low coverage of adsorbed target biomolecules (proteins, peptides, DNA, RNA *etc.*) within a reasonable time; this quantitative detection should preferably take place from small sample volumes.

Perhaps the most efficient method to approach this goal is to use fluorescent labels to enhance the signal [1]. A fluorescent molecule can emit thousands of photons before it bleaches, and even conventional detectors today approach single photon sensitivity. As a consequence, fluorescence-based detection schemes are often orders of magnitude more sensitive than the most common label-free surface-based techniques. The latter typically rely on detecting relative changes in average properties such as optical or mass density, such as for example surface plasmon resonance (SPR) [2], quartz crystal microbalance (QCM) [3], or electrochemical impedance techniques EI [4]. However, attachment of fluorescent or other chemical labels adds significant preparative steps and for complex biological systems, inhomogeneous labeling is often an issue of great concern. The labeling of molecules from native samples is most often also not possible. A standard and efficient way of circumventing the lack of labels on native samples is to use a sandwich assay in which one surface-immobilized antibody captures or detects an unlabeled target molecule (antigen) in a complex biological sample; a second antibody carries a marker, e.g., a fluorophore, which through binding directly to the capture antibody allows detection. Unfortunately, although it allows detection and it also may help increase the molecular specificity, the use of sandwich assays precludes recording of accurate binding kinetics. Without kinetic information both concentration and binding affinity determination become significantly more inaccurate.

In certain cases, external labels may also alter the nature of the interactions. For these reasons, but also because of their potential to extract detailed information on interaction kinetics, label-free surface-sensitive methods, such as QCM, EI and SPR have increased in popularity. Today, significant efforts are focused on developing miniaturized versions of such and related transducer principles for sensor applications. The primary advantages which are desired by miniaturization can be summarized as:

1. large scale multiplexing via multiple sensor elements on the very same chip;
2. handling of minute sample volumes by integration with micro-fluidics; and
3. (possibly) increased sensitivity and decreased limit of detection.

**Figure 1.**
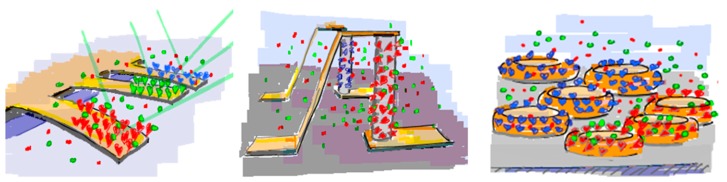
Schematic illustration of (**left**) small-scale surface-stress sensitive cantilevers, (**middle**) semiconductor nanowires and (**right**) nanoplasmonically active gold discs designed with immobilized probe molecules for selective detection of suspended analyte molecules. The different colors represent capture agents for different molecules immobilized on the respective sensors.

Promising miniaturized sensor principles include resonating or surface-stress sensitive cantilevers [5–10], semiconducting nanowires [11–16] and plasmonic nanostructures [17,18], which schematically are shown in Figure 1; these sensor configurations are analogous to the macroscopic counterparts QCM, EI and SPR, respectively.

Each technique has its own pros and cons, and the emerging consensus is that the choice of “best” sensor should be based on the desired application. For example, semiconductor nanowires sense changes in interfacial charge [13,14], which makes them best suited to probe highly charged molecules, such as DNA, that strongly increase the charge accumulation close to the sensor surface or charge translocation across cell-membrane mimics. One additional advantage is the simplicity by which changes in the electrical resistance of the nanowires induced by the change in interfacial charge can be measured. Nanowire sensitivity is improved by operation in aqueous solutions with low ionic strength. This means that operation under physiological conditions decreases the performance, which in turn limits their generic applicability. However, nanowires might be highly suited in new drug-screening assays to probe, e.g., membrane-protein controlled ion-translocation across supported lipid bilayers [12,15,19,20].

Small-scale cantilevers are primarily sensitive to changes in surface stress. Hence, in analogy with QCM, it is not straightforward or even obvious how to approach quantifying the measured response in terms of number of bound molecules. A linear response versus biomolecular surface density is necessary to analyze binding kinetics, which is the goal of most biosensors. Small-scale cantilevers might lack this ability but can instead, in analogy to QCM, provide unique information on structural changes in the adsorbed film of biomolecules [21]. This type of information is achieved by analyzing additional information such as energy dissipation from an oscillating cantilever and/or modeling the stress distribution. This approach has indeed emerged as a promising tool to probe structural changes of adsorbed biomolecules in response to their interaction with e.g., drug candidates [22,23].

Nanoplasmonic sensors share most of their sensing features with conventional SPR. The most important shared trait is that the measured response is, to a good approximation, linearly proportional to the amount of adsorbed analyte; this makes nanoplasmonic sensors well-suited to the analysis of binding kinetics. They are also ideally suited to determine the bulk concentration of analyte molecules if the dependence of the adsorption rate (or the amount of adsorbed molecules) on bulk concentration is known [24] Nanoplasmonic devices combine these properties with comparably high sensitivity in terms of lowest detectable surface coverage [25]. Nanoplasmonic sensors display a lower sensitivity to changes in bulk refractive index due to almost one order of magnitude shallower sensing depth compared to conventional SPR sensors; the sensing depth is on the order of tens rather than hundreds of nanometers. However, this can be used to increase their sensitivity since a larger part of the sensing volume is consumed by the sample probe. On the flip side this also limits their applicability to studies of large molecules and thick molecular (>10 nm) films. The shallow evanescent field can also be turned into another advantage, since redistribution of mass within the decaying field enables biomolecular or supramolecular structural alterations to be probed [26,27]. Combinations of evanescent field based sensors with responsive brushes and hydrogels have also been proposed and tested [28,29]. A responsive coating can be used to bring a larger amount of captured analyte into the sensitive region and thereby use the high localization of sensitivity to its fullest [28]. An additional advantage of nanoplasmonic sensors is that they are decidedly easy to miniaturize by many approaches, both as surface-based sensors and as bulk particle-based sensors [18,30]. This desirable combination has led to nanoplasmonics being the most popularly implemented miniaturized bioanalytical sensor platform.

There are numerous excellent reviews [4,11,31–34] and books [30,35,36] on these and additional types of transducer principles for label-free sensors available; we urge the interested reader to consult these reports for details on how to apply the respective transducer principles to obtain maximum biosensor sensitivity in different configurations. However, we recommend the reader to carefully evaluate the definition of sensitivity made in the literature, which in the case of biosensors should be stated as limit of detection (LOD) in terms of bulk concentration of target molecules. Consequently, the best comparison of different types of surface-based biosensors is, as argued above, the detection limit in terms of mass per unit area combined with the time required to reach LOD for a given concentration.

**Figure 2.**
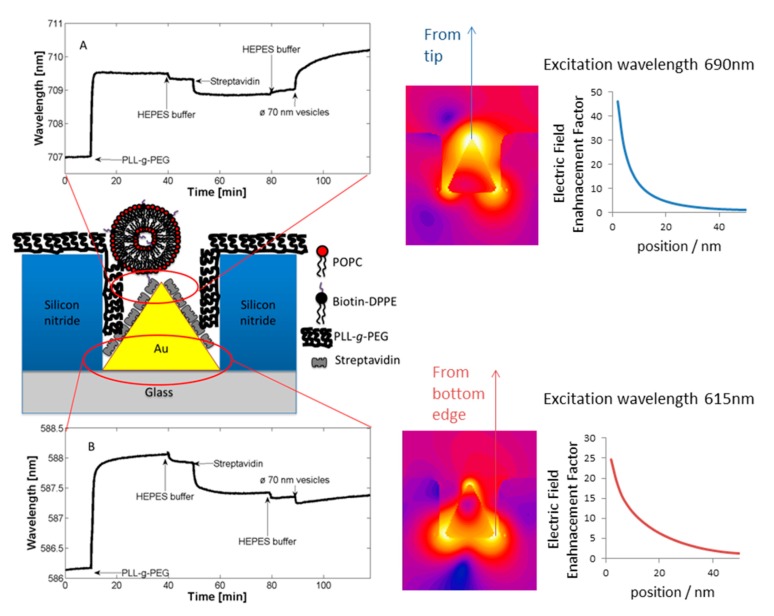
Example of the higher sensitivity at the edges of nanoplasmonic sensor elements. A 100-nm gold cone embedded in a nanocavity display the highest enhancement and measurement sensitivity at the base and the apex. The geometry was used to selectively measure molecules interacting only at the tip or at the base of the sensor element [38]. (**A**) Shows the sensor response at the apex and (**B**) shows the sensor response at the base. Simulations (right) show the highly localized electric field enhancement that explains the localized sensitivity.

The transducer principles discussed above have in common that they are based on converting a contrast in a physical property created by biomolecular recognition occurring at the sensor surface into a readily measurable signal. The physical properties used for this contrast are, e.g., mass, refractive index or electrical resistance. A time-resolved, quantitative measurement of molecular recognition can be recorded by performing the measurement of the contrast change at sufficient (sub-second resolution) speed. Macroscopic sensor elements can be approximated as having on average uniform sensitivity thanks to a physical extension that is much larger than their nanoscale sensing depth and the molecular dimensions of the analyte. However, the transducer becomes susceptible to edge effects as the sensor is miniaturized to nanoscale dimensions; this is a consequence of the finite lateral extension of the sensor element that approaches the length scale of the physical phenomenon used for the transducer principle, e.g., a plasmon polariton, mechanical wave or charge double-layer. The sensitivity across the sensor element can therefore vary by orders of magnitude [37]. Not uncommonly, the sensitivity is highest at the edges and corners of sensor element (*cf.* Figure 2) [38]. This is in particular true for nanoplasmonic devices but tends to be a general feature of nanodevices exploiting localized optical or electrical fields.

While the transducer principle is the most commonly discussed aspect of biosensors in the literature, in this review we will discuss a less often considered, but equally important, aspect that must be considered to optimize the performance of surface-based bioanalytical sensors: specific surface modification of the sensor element. The importance of proper surface modification, such as suppressing non-specific binding and introducing optimal ligands for specific binding are well recognized and apply to all sensor designs. However, it is more rarely discussed that there are additional constraints and extreme demands placed on small-scale sensors to detect low-abundant biomolecules present in a complex background of other biomolecules in biological fluids. This is the measurement situation encountered in biomarker identification [39–41] and disease diagnostics [42–45] to just name two important applications. In other words, the target molecules must bind with high molecular specificity to recognition (probe) elements on the sensor surfaces.

The level of specificity of the immobilized recognition element to the suspended target is defined based on the level of selective recognition of unique target molecules from a complex molecular mixture; high specificity thus requires choosing recognition elements such that cross-reactivity is low. The improved molecular selectivity obtained using multiple recognition elements is an additional reason why a secondary antibody is helpful in clinical diagnostic applications. It is also important that the immobilization preserves the 3-D structure of the recognition element since specific interactions of biological molecules are strongly dependent on 3-D conformation and binding geometry; such requirements can only be met by ensuring suitable surface (bio)chemistry on the sensor.

Selectivity of the interaction is in this context defined as the level at which only the desired target molecule binds to the surface, *i.e.*, high selectivity requires that the sensor surface is designed such that it is inert for all other proteins present in the sample. This does not only put strong requirements on high molecular specificity, but the underlying sensor surface must be designed such that it is inert to non-specific binding. It is here worthwhile to emphasize the importance of also keeping all surfaces other than the actual sensor surface, *i.e.*, the walls of liquid reservoirs, tubing and measurement chambers, inert towards protein adsorption. Non-specific protein adsorption to any surface will lead to reduced bulk concentration and skew both measurement and detection. This consideration should be even more strictly applied to small-scale sensors than to macroscopic sensors, since the sensor elements of the former comprise only a small fraction of the total surface. In addition, miniaturized sensors are generally composed of two or more materials. The interaction of the analyte should be confined only to the sensitive part of the miniaturized sensor to ensure detection, which corresponds to one of these materials.

When functionalizing the sensitive area of a sensor one is often, but not always, bound to a certain set of surface chemistry tools defined by the material comprising the sensitive element. For example, the sensitive part of a semiconducting nanowire sensor is typically an oxidized semiconductor and cannot be substituted for a metal or for a freely chosen other oxide. In contrast, small-scale cantilevers are compatible with essentially all substrate materials that can be deposited as a sufficiently thin film. Thus, while nanowires restrict the options, the flexibility is high with respect to the choice of surface modification for cantilevers. Nanoplasmonic sensors are restricted to metals, most often gold. Due to their shallow evanescent field, the sensitivity is compromised if another material is deposited on top of the metal [46] and the plasmonic properties change also if it is covered with or substituted for another metal.

The aim of the following sections is to provide an overview of: (i) which surface modification concepts are applicable to which sensor material; and (ii) how they can be combined together to achieve the desired different-orthogonal-functionalization of the sensor element and the surrounding surfaces. It may seem relatively straight forward to achieve these goals given all the surface functionalization tools that have been developed in the past decades. Unfortunately, as the literature reveals, in practice it is not. In this review we emphasize an understanding of the relevant design principles and tools over presenting a complete survey of all the literature on this vast topic.

## 2. Surface Modification Using Molecular Self-Assembly

**Figure 3.**
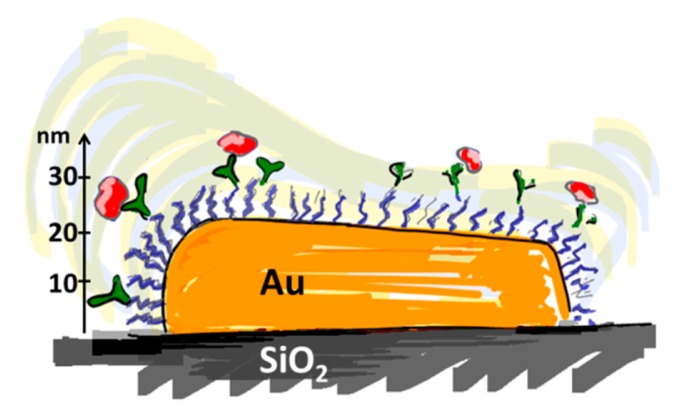
Schematic illustration of the relatively similar size of sensor element, surface coating, ligand and the sensitive region of a nanoscale sensor. A nanoplasmonic particle is used as example. A typical surface coating functionalizing the sensor might consume the greater part of the highest sensitivity region of the sensor element. A random distribution of functional groups might not correspond to the localized sensitivity of the sensor element, here, e.g., at high aspect ratio parts of the structure.

The ideal biosensor consists of bioactive areas coinciding with the sensitive areas of the sensor and inert areas everywhere else to ensure minimum sample consumption. The goal is to obtain the highest sensitivity, specificity and selectivity that is suited for the application. Both the sensor element itself and the surrounding surfaces thus have to be rendered inert to non-specific adsorption. This is a prerequisite for restricting binding of target molecules to the sensitive areas of the sensor by subsequent specific functionalization. To meet this challenge a functionalization scheme has to be developed that makes use of molecular patterning over the sensor. It is especially demanding in the case of small-scale sensors to achieve restricted patterning of functional molecules; the dimensions of the molecules used for passivation and capture as well as the analytes of interest are of a size comparable to the sensor element or at least comparable to the size of the sensitive area (Figure 3).

### 2.1. Boundary Conditions for Choice of Patterning Strategy

In deciding on a strategy to modify a particular sensor surface one must consider the substrate surface properties, the environmental conditions under which the coating must be stable (time frame, pH, storage conditions, *etc*.) and the degree of passivation to non-specific biomolecular interactions that is required. While the environmental conditions for application and storage might not differ greatly for biosensors, the materials which have to be functionalized, as well as the sensor geometry and the complexity of the analyte solution can vary greatly. For example, the type of solution to analyse can vary from controlled laboratory buffers containing a single biomolecular species to blood samples or sea water samples with high molecular complexity. A greater number of different kinds of molecules, a higher concentration of molecules and higher molecular weight molecules in the sample make it more difficult to prevent non-specific adsorption. Likewise, prolonged storage or the need to operate the sensor over a wide range of pH, temperature or ionic strengths might deteriorate a molecular surface coating. As the coating deteriorates varied types of non-specific adsorption can occur.

**Figure 4.**
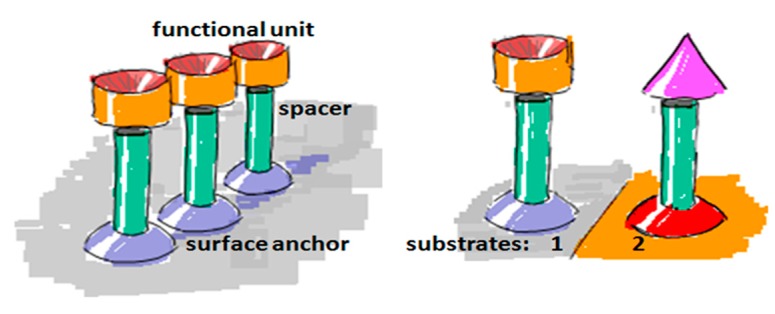
Schematic illustration of the different molecular components required to create a biosensor surface. The surface anchor ensures the binding to the underlying substrate material, the spacer's role is to screen all interactions of the target with the substrate, and the optional functional unit (recognition element) is used to selectively capture the target. (**Left**): A surface modification scheme on a single substrate material, representative for macro-scale biosensors; (**Right**): A biosensor surface consisting of two different substrate materials, as often encountered in small (nano) scale sensors. Two different anchors, each specific to one of the substrate materials, are required to modify the substrate.

To create a non-fouling interface it is instructive to divide the design of the molecular interface into three mostly independently designable parts (Figure 4): anchor, spacer and functional unit (recognition element). The role of each component will be discussed below with particular focus on the design criteria for each of them to generate a biofunctional pattern.

### 2.2. Surface Anchor: How to Define and Retain a Molecular Pattern

The surface anchor is perhaps the most decisive choice to design a molecular interface for both macroscopic and nanoscale biosensors. The choice of anchor strategy determines the stability of the molecular interface, as it determines how strongly attached the molecules are—which for a choice of anchor can vary depending on the environmental conditions of the measurement. Further, certain anchor strategies will enable transitions of the molecules at the sensor interface, including in the mobility of functional units; other choices impose a fixed organization. The choice of anchor in concert with the spacer also determines the achievable packing density of both spacers and recognition elements.

An anchor has to be chosen according to the above design criteria while taking into account the materials chemistry of the sensor element itself. Most choices are specific to the material it has to bind to and cannot be generally used. For a miniaturized sensor the sensor element will be made of a different material than the surrounding surface. This suggests the use of materials specific molecular binding groups as anchors; then, functional units can be bound exclusively to the sensing element and a non-fouling molecular coating can be bound to surrounding areas. Anchors that bind specifically to the material of the sensor element but not to the background attach functional units only to the sensor element by self-assembly. A second type of anchor specific to the surrounding surface chemistry can then be used to backfill the surface with a second molecular coating; this coating in turn should suppress binding of additional molecules. Nonspecific binding to the background should be suppressed completely close to the sensor element since it can influence the signal. Away from the sensor element nonspecific binding can cause depletion of the bulk concentration and reduced transport to the sensor element, but the requirement on suppressing binding to the these parts of the sensor is less strict. The use of anchors with such orthogonal binding properties to produce molecular patterns by molecular self-assembly was first demonstrated by the Whitesides group [47], and has since been repeatedly used. Examples of sensors with materials contrast suitable for orthogonal molecular self-assembly through the anchor chemistry are nanoplasmonic gold sensors on glass (SiO_2_) background [48,49] and metal nanowires or carbon nanotubes suspended over silicon oxide or silicon nitride [34,50]. Often the materials contrast is between a metal and an oxide, e.g., glass/SiO_2_, background, but a sensor element can also itself be an oxide or coated with an oxide such as TiO_2_ or ITO [51,52].

Strategies for anchoring can be discussed from several different starting points, but one approach is whether the anchor attaches strongly enough to be comparable to chemisorption or whether weaker physical interactions like electrostatic or dipolar interactions are made use of.

### 2.3. Weak Anchors—“Physisorption”

A single weak bond, *i.e.*, an interaction not forming a chemical bond, provides a finite residence time at the interface for its bound molecule; the residence time is likely to be much shorter than the time scale of the measurement for a significant fraction of the molecules coating the surface. Such bonds are the norm for biological recognition and can be quantified through the equilibrium constant, which is the target of many biosensor measurements. However, the surface coating enabling recognition measurements must be stable over the entire time scale of the experiment. Thus, multiple anchor units have to be linked to each spacer for it to remain at the interface if weak interactions are used to anchor the spacer and functional units to the sensor surface. If this is not the case, non-specific adsorption to the sensor area will occur when defects in the coating are created by desorption of spacers. Further, if functional units are reversibly anchored, the number of active functional groups at the interface will fluctuate and decrease with time.

A commonly used strategy is therefore to increase the anchor size in order to allow multiple coupled but weakly adhering anchor units to bind to the sensor substrate simultaneously. If one unit reversibly comes off, it will remain in the vicinity of the interface for a sufficiently long time to rebind since it is restricted by the remaining units still bound to the substrate. A dramatic increase in the binding affinity of macromolecules can thus be achieved [53]. The simplest example of this strategy is the use of proteins like albumins to render surfaces non-fouling. The single amino acid affinity to the surface is very low, but the multiple contacts of peptide sequences on the protein results in, for practical purposes, irreversible protein affinity to the surface through a very large number of attractive, mainly van der Waals, interactions.

Naturally, more advanced concepts have evolved that allow a higher degree of interfacial control and even substrate specific adhesion. Probably the most well-established such strategy is the poly(L-lysine)-*graft*-poly(ethylene glycol) (PLL-*g*-PEG) platform [54,55]. Multiple poly(ethylene glycol) spacer chains are grafted along the backbone of a highly charged polylysine chain. This anchor architecture allows many weak attachment points through the backbone onto which a desired density of spacer side chains can be added. The spacer and functional units can thus also be varied by synthesis and be incorporated into a single macromolecule at a desired ratio [55]. These macromolecules adsorb on negatively charged surfaces, e.g., most oxides used for biosensors. By design, they provide a precise ratio of tailored spacers and functional units to the surface. Other examples of similar strategies using graft or block copolymers are PSS-PEG [56], PEI-*g*-PEG [57,58] and PLL-*g*-PMOXA [59,60] which rely on charge interactions, and poly(ethylene oxide)-poly(propylene oxide)-poly(ethylene oxide) (Pluronic^®^) [61] which relies on one block having affinity to hydrophobic interfaces. Typical backbone sizes are several tens of kDa while side-grafted blocks required for surface passivation to protein adsorption should be at least several kDa.

A great effort has been invested to find the optimal relation between the number of monomer units on the backbone that are used to anchor the macromolecule to the substrate and the fraction that are used side-graft the spacer. Furthermore, there is a compromise between the ideal molecular weight of the spacer, its grafting density along the chain and the overall molecular weight of the macromolecule. The thorough optimization of these anchor strategies has led to very successful protocols to render surfaces non-fouling or to give them specific functionality. The suppression of nonspecific binding of proteins from, e.g., serum and other protein solutions has suggested that a very high surface density of spacer units and low density of defects can be achieved [62,63]. A possible reason for this good performance is that the reversibility of binding of individual anchor units along the backbone chain due to weak anchoring allows rearrangement and high packing of the anchored units on the surface. This clever design allows both high overall surface affinity and sufficient lateral surface mobility to increase packing density beyond what can be achieved by random adsorption. A chain of multiple anchors each with high surface affinity will instead freeze the system in a non-densely packed conformation that can even include loops of the backbone. Such a surface modification would leave both a high surface density of defects and of exposed backbone open to non-specific binding [64].

Despite this significant advantage, the strategy of using multiple low-affinity anchors per macromolecule also can have drawbacks. Consider what happens when charged functional groups with similar affinity to the substrate as the anchor groups are used; then, the functional groups might compete favorably for adhesion sites at the interface. At sufficient density they will disrupt the desired orientation, conformation of the layered coating as well as reducing the density of functional groups available for binding at the interface. Similarly, hydrophobic functional groups can lead to micelle formation in the bulk. The weak subunit surface-affinity might not be sufficient to break up the micelles during surface assembly to form a well oriented molecular interface.

Finally, it is sufficient that only part of the physisorbing anchor backbone adsorbs to the surface to ensure strong surface adhesion; the remaining parts of the backbone which are not bound e.g., due to space constriction by neighboring surface-bound macromolecules or a different surface chemistry are then forced to extend out into the solution. Such exposed backbones are likely to always be present for kinetic reasons, but the resulting defects are not dense enough or providing sufficient binding affinity for noticeable fouling of small extracellular proteins. However, their presence has been indicated for example through binding of larger objects, e.g., by measurable fouling of negatively charged liposomes (∼100 nm in diameter) to PLL-*g*-PEG while zwitterionic liposomes did not bind [65,66].

In the typical example of PLL-*g*-PEG, the adsorption is achieved through electrostatic interaction of a positively charged backbone with a negatively charged substrate surface. The total charge interaction overcomes the entropic and other energy contributions that drive the macromolecule to desorb into solution again. It also ensures the directionality of the anchor-spacer-functional group, which is necessary to control the interfacial properties. While charge is not a materials specific interaction, it can nonetheless be used to pattern sensors. This can be achieved by direct self-assembly on substrates prepatterned with e.g., an oxide and a metal, as is the case for many nanoscale sensors. Metal oxides and semi-metal oxides for biosensor applications mostly produce surfaces with a strongly negative surface potential at physiological pH, while gold which lacks a thick and stable surface oxide produces only a weaker mirror charge interaction. Strong binding will then only be achieved to the oxide while weakly anchored macromolecules can be washed off or replaced by more strongly adsorbing species on the gold surface [67]. Alternatively, material specific chemisorption to e.g., gold is used prior to back-filling using, e.g., PLL-*g*-PEG [49], as will be described below.

### 2.4. Strong Anchors—“Chemisorption”

Chemisorption in the field of surface functionalization typically does not imply the formation of a full covalent bond. In practice, this term has tended to include anchor strategies that produce bonds of lower strength than typically acknowledged as a chemical bond, but that use stronger charge-type and electron-sharing interactions to immobilize a molecule over long time scales at the interface. A binding strength of an individual anchor sufficiently high to irreversibly bind a spacer to the surface over the life-span of a sensor allows tethering of spacer and functional units using single anchor moieties. The most prominent examples of such anchors are silanes (R-Si(OH)_3_), which bind to e.g., silicon dioxide, and thiols (R-SH), which bind strongly to certain metals, such as gold, silver and copper. Historically, these binding mechanisms have been explored in the context of the development of self-assembled hydrocarbon monolayers (SAM) in the 1980s [68,69]. They have been amply applied since then to modify sensor surfaces and nanoparticles with longer and bulkier polymers such as PEG [70,71] and DNA [72]. The same holds for phosphate (R-OPO(OH)_2_) and phosphonate (R-PO(OH)_2_), which bind to e.g., TiO_2_ and iron oxides [73–75]. Recently, anchors such as catechol derivatives, inspired by marine animal adhesion and metal fixating microbes, have also been used as high affinity anchors to metal oxides [76]. More examples exist, but in relation to the large number of possibilities for (bioinspired) chemical coupling, the number of well characterized anchor systems for molecular coating of common sensor materials must be considered rather limited.

Characterization of the quality of the formed coating and of its durability becomes particularly important for patterning applications, because at least two different anchor chemistries with potentially high surface affinity will compete. Successful patterning also requires a detailed understanding of the molecular coating process itself. Patterning through molecular assembly of chemisorbing anchors utilizing materials contrast of the sensor elements to the surrounding areas requires anchors with orthogonal binding affinities to these materials. That is, one anchor should bind to one material with high enough affinity to ensure irreversible tethering of spacers and functional elements, while its affinity to other areas should remain sufficiently low to be replaced by another anchor with irreversible affinity to that surface material. Since the interactions are chemical in nature, such orthogonality is expected to be found. For example, while thiols bind strongly to gold they have very low affinity to oxides [47]. Silanes on the other hand have very low affinity to gold. Sequential adsorption of different species tagged with the respective anchors would therefore translate the underlying sensor materials contrast into molecular patterns at the interface. The transfer of a substrate materials contrast to a self-assembled molecular contrast is sometimes referred to as selective molecular assembly patterning (SMAP) [77]. Recently, nanoplasmonic devices were functionalized in up to three steps using this type of approach [78].

**Figure 5.**
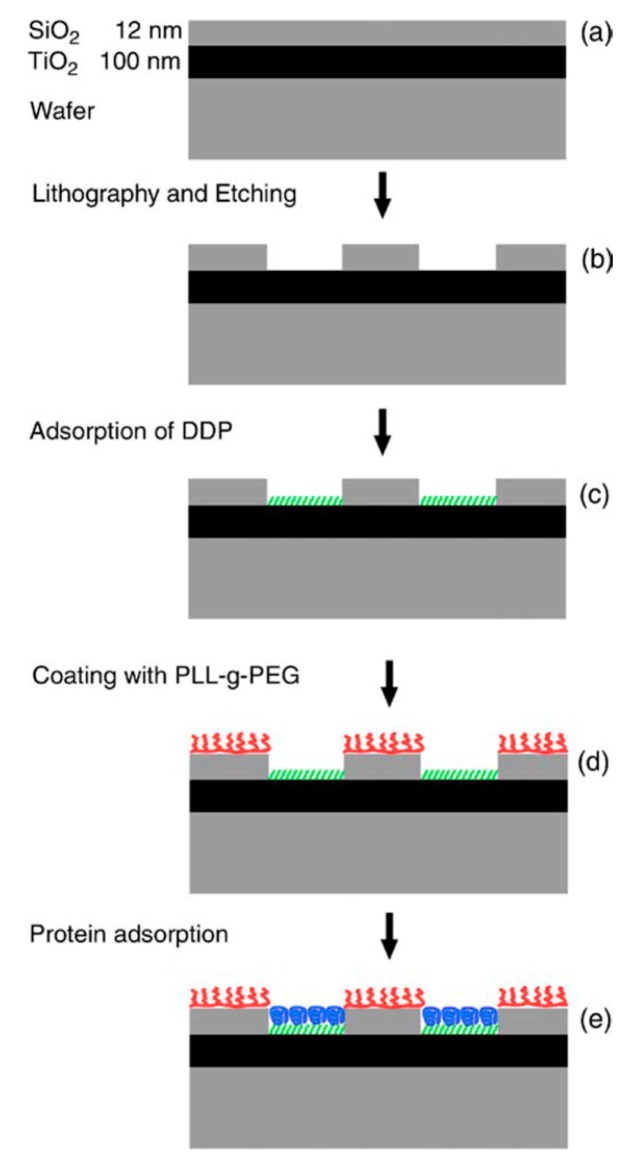
Schematic illustration of the original SMAP method [79]. Material contrast created in oxides by lithographic techniques is converted, in a series of dip-and-rinse processes performed in aqueous solutions, into a contrast with respect to protein adsorption: (**a**) a polished silicon or transparent glass wafer is coated first with a 50 nm titanium oxide intermediate layer and then with a 12 nm thin silicon oxide top layer; (**b**) The desired patterns are created in the metal oxide layer by a combination of lithographic and etching processes; (**c**) Adsorption of dodecyl phosphate (DDP) from aqueous solution leads to the formation of an oriented self-assembled monolayer on TiO_2_, making it hydrophobic. There is no interaction between DDP and the SiO_2_ surface, which is left completely bare; (**d**) After rinsing with water, PLL-*g*-PEG adsorbs from a buffered solution to the bare SiO_2_, and to a lesser extent also to the DDP (not shown). After rinsing, the PLL-*g*-PEG-coated SiO_2_ regions repel proteins completely, while the PLL-*g*-PEG adsorbed on the DDP is weakly bound and is exchanged with the adsorbing protein(s); (**e**) The chemical contrast between hydrophobic and protein-resistant areas can then be converted into an adhesive/biofunctional contrast by simply exposing the surface to proteins.

This approach can be taken to even higher complexity by using some of the other mentioned anchors. Phosphate and phosphonates bind with high specificity to TiO_2_ over SiO_2_; this specificity has been used for molecular patterning down to the nanoscale using phosphonate anchored self-assembled monolayers and proteins on a PEG background (Figure 5) [77].

However, although this selectivity is good, the affinity of phosphonates to TiO_2_ is low and therefore not sufficient to tether, e.g., non-fouling polymer brushes such as PEG. More useful in this respect are catechol derivatives such as the well-known dopamine [80,81] and DOPA [81–83], or the more novel nitrodopamine [81,83,84], nitroDOPA [81,83,84] and anacat [81]. These anchors have recently been investigated and found to yield very high affinities to oxides such as TiO_2_ and Fe_3_O_4_, but low affinities e.g., to SiO_2_, oxidized gold and Fe_2_O_3_ [75,81,84]. Therefore, they are more promising candidates relative phosphonates (too low affinity) and silanes (low specificity and difficult to pattern due to sensitivity to trace amounts of water) to be used for orthogonal patterning on the nanoscale.

### 2.5. Weak vs. Strong Anchors for Nanoscale Sensors

Which anchor strategy should be chosen for patterning nanostructured sensors in order to maximize the capture efficiency of analytes at the part of the sensor with the highest sensitivity? The literature on the subject of nanoscale sensor functionalization is not able to deliver a final answer to this question yet. It is likely that the answer will depend on the specific material and geometry of the sensor. A few considerations based on comparing the differences between “chemisorbed” single anchors and “physisorbed” multiple anchors can however be enlightening for further investigations into this subject (see Table 1).

**Figure 6.**
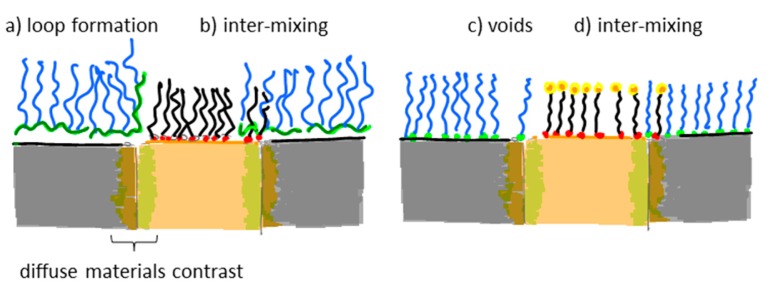
Schematic of materials selective surface functionalization at the interface between two materials. A nanoscale region where the materials contrast is not ideally defined can be created by interdiffusion of the materials or by an additional bridging material (e.g., adhesion layer). The surface functionalization is then likely affected in any of several ways: for multivalent weak anchors (**a**) loop formation producing charged or hydrophobic defects can occur; (**b**) intermixing and a diffuse biochemical contrast can be created; for chemisorbing single anchors; (**c**) voids due to insufficient binding can open the substrate to nonspecific binding (can also occur for multivalent weak anchors); (**d**) intermixing and a diffuse biochemical contrast can be created.

Many nanoscale biosensors, e.g., nanoplasmonic sensors [85], have high sensitivity at the border region between the sensor element and the surrounding substrate. Depending on the fabrication method this region can also show some inter-diffusion of the different substrate materials. Furthermore, nanometer-thick adhesion layers often are used to make metals adhere to oxides. These non-ideal conditions lead to specific problems with the respective functionalization strategy (Figure 6) [85]. Chemisorbed single anchors will for non-intermixing sensor substrate materials in principle allow for perfect definition of the sensing element and the background using orthogonally binding anchors. However, if there is intermixing of two or more materials in the substrate, e.g., Ti in the adhesion layer diffusing into the gold, then thiols used to specifically functionalize the gold sensor might show reduced affinity in the border region (where the sensitivity of the sensor is high). Reciprocally, nitrocatechols with high affinity to a TiO_2_ surrounding background might bind also to the edge of the sensor element (Figure 6d). This complication can also be used to an advantage. For instance, the non-specific binding could be reduced by almost one order of magnitude (from a few percent to less than one percent) for nanoplasmonic gold hole sensors entirely surrounded by the same material as used for adhesion of gold, *i.e.*, TiO_2_ [85], compared to the same sensor design with the gold adhered to SiO_2_ using Cr as the adhesion layer to a SiO_2_ background [49].

Flexible and reversibly adsorbing macromolecules such as PLL-*g*-PEG might bridge areas of ill-defined surface chemistry. They will display some affinity to an inter-mixed materials region and due to their size they might extend over it with sufficient attachment points to the oxide to which they adhere well. However, the large size also means that they can extend onto e.g., an electrode or plasmonic sensor area from the passivated background even when this is not desired (Figure 6b). The conformation generally taken by physisorbed macromolecules at the border between materials to which they adhere with different affinities is not well investigated. In contrast to single high affinity anchors it is unlikely that macromolecules with multiple weak affinity anchors can create a well-defined boundary on the molecular scale between two materials. This is regardless of the assembly process since the anchor chain is free to rearrange after the initial coating; it is likely that some underlying substrate area or part of the charged or hydrophobic backbone is exposed to the solution (Figure 6a,c). Such defects will lead to non-specific interactions as discussed above. Thus, it is not clear that any of the outlined orthogonal patterning methods will always lead to the desired definition and suppression of non-specific binding at the edge of a nanoscale sensor. Each sensor pattern combined with a particular patterning method should be considered to risk significant background response at the most sensitive parts of the sensor.

In terms of flexibility of adding functionality to the surface, the two anchor strategies also differ. Using macromolecules with multiple anchors of low affinity allows synthesis of molecules that have multiple spacers and functional groups per molecule. Thus, the ratio of functional groups to spacers can be precisely defined and characterized before the assembly through chemical synthesis and analysis. The same ratio will always be obtained after self-assembly on the sensor. However, for each newly desired ratio or functional mixture, new macromolecules have to be synthesized. The self-assembly of each new macromolecule has to be characterized since the method is not modular in the assembly stage. Due to the typical many-nanometer-size of these molecules and the weak interactions used for the assembly, a simple mixing of differently functionalized macromolecules will lead to nanoscale heterogeneity in the coverage of functional groups on the sensor. The consequences of this are more or less difficult to predict for different sensor designs. On a nanoscale sensor the sensitivity is typically also unevenly distributed over the sensor and distribution of e.g., capture groups over the sensor therefore can strongly influence both timing and absolute magnitude of the response.

Libraries of different spacers and functional groups can be created in advance using chemisorbing single anchors [75]. If their molecular weights and solubility are reasonably similar, a simple mix and match approach can be employed by which the molar ratios of interest are mixed in the bulk to self-assemble into a similar ratio on the surface [86]. While phase segregation is well known in SAMs [87,88], this problem is generally believed to be minor for the assembly of differently functionalized polymer brushes. In the latter case the same magnitude of repulsive interactions should dominate the assembly also for heterogeneous mixtures. However, in practice, deviations of the surface molar ratio from the bulk molar ratio can be observed; replication of bulk ratios cannot be taken for granted [86,89].

### 2.6. Spacer: How to Suppress Binding

Anchors are the key to creation of patterns and the stability of the sensor architecture, but the choice of properties of the spacer unit will determine the effectiveness of the interface in controlling non-specific biomolecule interactions with the sensor. Additionally, the choice of strategy to attach the spacer to the anchor will influence the binding and assembly of the anchors to the surface. Simple, biologically derived spacers such as proteins and lipids can be used, as well as self-assembled monolayers. However, the by far most common spacers for engineered biosensor interfaces and the most suitable for patterning are hydrophilic polymers, e.g., poly(ethylene glycol) (PEG) (see Table 2 for an overview of different passivation strategies). The goal of the spacer is to completely screen the interactions of molecules in the solution to be analyzed with the underlying sensor substrate without introducing any new attractive or long-range repulsive interactions.

**Figure 7.**
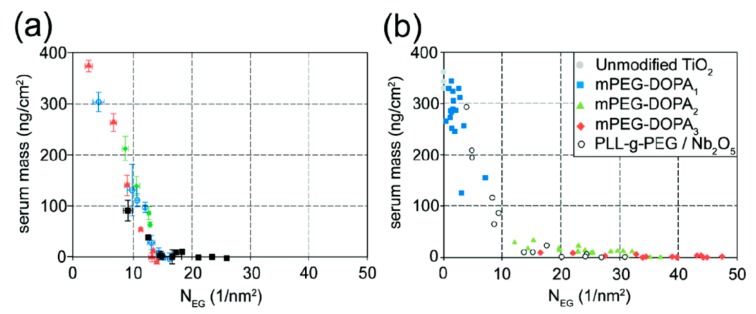
A “master curve” compiled for the efficiency of different anchoring strategies and PEG spacers with respect to preventing serum protein adsorption. A mono-, di-, or trivalent DOPA or a PLL-chain multivalent anchor was used. These combined results from several studies for short linear and dendron PEG show that serum protein adsorption is prevented above a cut-off projected surface density of ∼15 EG units (*N_EG_*) per nm^2^, regardless of architecture. (**a**) Comparison of dendron (D(valency of anchor)) and linear PEG (L(valency of anchor) with a molecular weight of the PEG part of M_w_ = 2.5 kDa): D(*n* = 1), green diamond, D(*n* = 2), red triangle, D(*n* = 3), blue circle, and L(*n* = 3), black square; (**b**) Comparison of PEG-5 kDa-DOPA (D(*n* = 1–3)) (on TiO2) and PLL-*g*-PEG (on Nb2O5) [93].

The water-like hydrogen bonding ability of PEG results in low affinity of water-soluble biological compounds. The entropic and enthalpic (for most hydrophilic polymers) contributions of the polymer coil upon compression ensure a high energy penalty for any molecule trying to approach the substrate surface [90,91]; thus, non-specific biomolecule adsorption due to short-range van der Waals interactions and longer-range electrostatic interactions can be prevented if the attached polymer brush is sufficiently thicker than the van der Waals interaction length and in particular the Debye screening length [75,91]. The thickness of a film of tethered linear polymer chains is predominantly determined by the Kuhn or persistence length of the polymer, the molecular weight and the grafting density of the polymer [92]. For a given (short) polymer this can be translated into a “universal curve” for monomer area density, for which non-specific adsorption is suppressed (Figure 7); the combination of molecular weight and grafting density is chosen on this curve to produce a monomer area density over a threshold value at which protein adsorption below the detection limit is observed [55,60]. As can be seen in Figure 7, the concept of monomer density per area unit as a guide to whether nonfouling is achieved or not seems to translate also to branched polymer spacers such as dendrimers [93].

A thicker spacer layer can in general be considered advantageous to suppress non-specific binding, which is obvious from the thumb rule of maximizing the monomer density per area unit as for a similar grafting density the thickness of the spacer layer and the energy penalty for polymer displacement will increase. However, for surface sensitive sensors a thicker layer sacrifices sensitivity; this is in particular true for nanoscale sensors such as nanoplasmonic devices. The sensitivity of many surface based sensors rapidly decays, often exponentially, with distance from the substrate. The spacer layer is not part of the sensor element and thus constitutes a dead volume where sensing does not occur. For example, nanoplasmonic devices and capacitive sensors are sensitive over a length scale on the order of the size of most commonly used spacer molecules. Minimization of the spacer thickness with retained suppression of non-specific adsorption is thus necessary. This could be taken as an argument to use more compact, branched hydrophilic spacers rather than linear spacers for nanoscale sensors. However, such spacers are less common and therefore less investigated in comparison to, e.g., linear PEG spacers.

Orientation of the spacer layer to ensure the orientation also of the recognition element and the bound analyte is also important to ensure sensitivity of certain types of nanosensors. Silicon nanowires lose sensitivity if the polarization of the molecules is randomized compared to when it is aligned [94]. Thus highly ordered coatings are favored for certain applications and have to be achieved according to the nanoscale symmetry of the sensor element.

### 2.7. Grafting from vs. Grafting to

**Figure 8.**
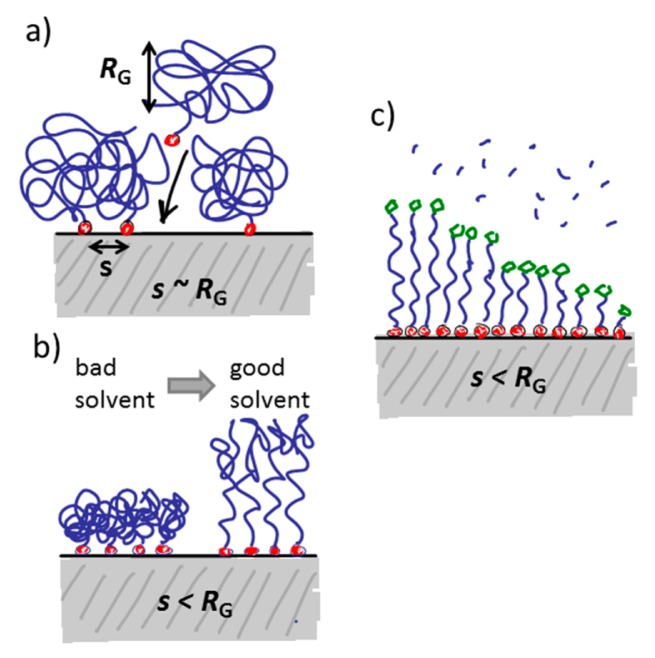
Schematic of “grafting to” and “grafting from” as standard techniques to create a sterically repulsive polymer coating at a sensor interface. (**a**) In grafting to a pre-synthesized anchor and spacer molecule is linked to the interface and the spacing between anchor groups (*s*) will at best on average be close to the radius of gyration of the polymer (*R_G_*). The polymer will be in mushroom configuration; (**b**) Grafting to can be performed in a poor solvent which reduces *R_G_* and allows for closer packing. Changing to a good solvent a polymer brush is created by swelling; (**c**) In grafting from an initiator is linked by an anchor to the surface. The polymer spacer is created by living polymerization from monomers in solution to create a dense brush.

There are two main strategies to form the spacer film typically referred to as “grafting from” and “grafting to” (Figure 8). The most straightforward strategy to produce large area films is the “grafting from” technique, by which selected anchors are first immobilized on the surface with initiators attached. The brush polymer spacer is then grown from the surface by an *in situ* surface-initiated polymerization (SIP) reaction. Monomers present in the solution get polymerized onto the surface by e.g., radical chain polymerization [95], living cationic polymerization [96] or controlled living polymerization such as atomic-transfer-radical-polymerization (ATRP) [97]. Both thin and thick films can be produced by these methods. Branched spacers can be created this way, but the monodispersity of the product is better controlled for linear chains. It should be noted that patterning with this method requires either patterning of the anchors in several steps with the polymerization on one part of the pattern in between each anchoring step and killing of all radical or living groups before the start of the next patterning step, or to find orthogonal sets of initiators where one initiator survives the polymer growth conditions of the first initiator. The latter is a challenge. Additionally, it is difficult to tune the fraction of functional units on the pattern by *in situ* growth of the spacer since every spacer will be identical and therefore the functionalization of the spacers is also typically 100%. Therefore, despite its advantage over the “grafting to” technique in terms of achieving dense polymer brushes (density determined by the initial initiator density in turn defined by the anchor foot print) it is not preferred for functionalization and patterning of sensors for which the distribution and density of functional groups is of prime importance.

In the “grafting to” approach, the spacer (and functional group) is chemically linked to the anchor before the latter is adsorbed onto the sensor surface. Therefore, the discussion of anchor patterning is possible to translate directly into the patterning of spacers and functional groups with the “grafting to” approach. The same level of definition can be obtained for the pattering of the functional group as for the anchor patterning. However, if a bulky spacer like a polymer brush is to be formed, the physical extension of the spacer and mutual exclusion interactions will determine the grafting density on the surface. This leads to a grafting density significantly below the polymer brush regime and therefore below the monomer density per unit area necessary to suppress nonspecific adsorption of most biomolecules. This drawback can mostly be circumvented by either adsorbing macromolecules like PLL-*g*-PEG for which the spacer grafting density is already sufficiently high from synthesis, or by adsorbing the anchor-spacer complex in a poor solvent for the spacer [75,81,86,98,99]. In the latter approach, the spacer will collapse to a fraction of its well solubilized volume in water and therefore allow a higher density to be achieved on the surface. When the sensor is introduced into the aqueous working solution the polymer spacer then swells and forms a brush. In this way, the density can mostly be made sufficient to suppress nonspecific binding [81,91,98]. Having to take spacer solubility into account during the anchor patterning stage is an additional complication during assembly and patterning. Optimization of the surface coating now entails that low solubility has to be balanced against homogenous coverage and sufficiently fast and efficient reaction kinetics for the binding to the surface.

In summary, the “grafting to” approach is more advantageous for grafting polymer brushes as spacers for most nanoscale biosensors, since for nanoscale structures it allows tailoring of the pattern exclusively through anchor orthogonality as discussed above. It furthermore, and importantly, offers mixing of anchor-spacer molecules through self-assembly to control the density and distribution of functional groups over the sensor surface.

### 2.8. Recognizing and Capturing Analytes on a Nanostructure

We have so far discussed how to successfully pattern functionality onto a miniaturized biosensor and to achieve this on a non-interacting background. Our remaining task is to design the functionality to perform its desired purpose of specifically and selectively capturing the analyte to the actual sensor element. A review of different recognition elements is not a focus of this section, but a few important points to consider when choosing the recognition element will be given. The in-depth interested reader is referred to any of the already existing reviews which address different techniques to couple biological recognition elements to solid substrates [100–102].

The first criterion is general for all biosensors that require capture of target molecules: the functional group has to provide a high affinity through common and weak physical interactions arranged on the surface of a macromolecule. A low affinity reduces the sensitivity of detection and this requirement is therefore analogous to the concept of sensitivity in clinical assays, *i.e.*, how well can we detect (capture) the presence of an analyte. The high affinity has to be achieved by many weak physical interactions; therefore, it can typically only be achieved by conservation of the 3-D structure of the immobilized probe complementary to the biomolecule (target) of interest. Since such recognition elements are beyond current state-of-the-art of organic chemistry, recognition elements are usually collected from libraries of existing or evolved biological compounds performing recognition functions in biological systems. Choosing recognition elements that already exist in biological systems also aids but does not guarantee that we fulfil the second criterion: specificity or selectivity. Again in analogy to clinical assays specificity refers to how many recognition events result from false positives. Selectivity is assured by avoiding both specific and non-specific attractive molecular interactions with other species that are present in the analyte solution. Selectivity is improved by arranging multiple weak interactions in a geometrical pattern that matches complementary molecular groups on the analyte since structure in addition to physical properties are then reflected. However, substantial affinity to other similar analytes can remain. In other words, even if a recognition molecule has a strong interaction with the analyte it might also have high affinity to another compound present in the sample; this would lead to low selectivity despite high affinity as the two species will compete.

The most commonly discussed, and used, biologically derived recognition elements are antibodies, which increasingly are being replaced by engineered fragments of antibodies (Fab fragments). Simpler peptides are also often discussed as recognition elements, but mainly used for targeting through e.g., cationic peptide interaction with negatively charged membranes or other similar supramolecular interactions. The single molecule affinity is low and therefore not ideal for nanoscale sensors developed to detect analytes at low abundance. Aptamers have received a lot of interest in recent years; they are a DNA-based alternative to peptides for creating specific recognition elements with defined 3-D structure. They also have a rather low affinity. We will briefly introduce these common systems and then discuss in more general terms important design criteria for adding recognition elements to biosensor surface functionalization schemes.

#### 2.8.1. Antibodies

Antibodies are widely used because of the high specificity of the antibody-antigen binding. However, they are also very sensitive to their environment, meaning that the way they are immobilized should carefully take into account that the correct conformation and orientation are achieved. Immunoglobulin G (IgG) is the most commonly encountered antibody in biosensing. It consists of one Fc and two Fab domains, and it is required that at least one of the antigen-binding Fab fragments are exposed to solution [103]. Immobilization techniques have included microcontact printing [104], biotin-streptavidin binding utilizing biotin-modified IgGs [105–108], direct spotting [105] and covalent binding [109].

#### 2.8.2. Antibody Fragments

Antibody fragments provide the same specificity as whole antibodies, but by having a large part of the antibody omitted they are much smaller. The Fab fragments are created by enzymatic cleaving off from the Fc fragment; the removal of the Fc fragment reduces nonspecific binding and improves selectivity. In the context of sensors with the sensitivity closely confined to the surface, such as semiconductor nanowires and nanoplamsonic sensors, this is a key advantage over antibodies. Orientation of an antibody fragment to expose its binding site is essential and somewhat more demanding to achieve than for antibodies due to that the Fc fragment cannot be used as the anchoring point. Up to a three-fold increase in activation has been reported [103] when the orientation has been controlled compared to random immobilization at the sensor interface. Common immobilization strategies include binding to streptavidin coated surfaces by biotinylation [106] and direct coupling to gold using reduced cystein residues [110].

#### 2.8.3. Aptamers

Aptamers are single-stranded DNA or RNA oligonucleotide sequences folded into a three-dimensional structure capable of binding specifically to a target molecule. They are generated in the SELEX (systematic evolution of ligands by exponential enrichment) process which was first reported by Ellington [111] and Tuerk [112]. Suitable binding sequences are first isolated from large oligonucleotide libraries and subsequently amplified [113,114]. Aptamers are characterized by both relatively high affinity and specificity to their targets [113,114], but are more resource intensive to develop with somewhat lower affinity than antibodies. Despite being similar to antibodies in function, their quasi-synthetic origin offers several advantages such as more easily engineered coupling to sensor surfaces, higher reproducibility, longer shelf-life, easier regeneration and a higher resistance to denaturation.

### 2.9. General Considerations for Recognition Element Immobilization

As already described in the section on anchors, immobilization of a recognition element to the spacer layer and the underlying sensor element has to be irreversible. Surface density, conservation of active conformation and optimum orientation of recognition units such as proteins/antibodies or enzymes are additional challenges [115,116]. Although the surface density of recognition elements will determine the maximum attainable sensor signal, simple maximization of the density is not advisable; steric hindrance as well as denaturation due to mutual strong interactions between recognition elements can occur and lead to inactivity [103].

Proper immobilization onto the designated part of the pattern is important; it is a prerequisite for both affinity and selectivity. The structure of the recognition element has to be preserved to ensure specificity. Denaturation of the specific conformation also risks causing reduced selectivity by exposing non-specific binding domains. Performing the functionalization from, e.g., a polymer surface designed to reduce nonspecific binding mostly ensures that the surface does not strongly affect the 3-D structure of the recognition element. However, the orientation of the recognition element also has to be such that binding can occur [117]. Orientation of molecules at biosensor interfaces, especially at nanostructured interfaces, is difficult to verify and mostly one has to rely on the knowledge that the coupling elements have been designed such that the correct orientation is achieved during self-assembly.

#### 2.9.1. Biotin-Avidin Coupling

A common coupling strategy in lab applications that deserves special attention is the biotin-avidin coupling. A biotinylated nonfouling surface such as PLL-*g*-PEG-biotin is exposed to an avidin derivative with low nonspecific binding, such as streptavidin or neutravidin. The avidins have multiple biotin binding sites and adsorb as a top layer which can bind additional biotinylated ligands through their free binding sites [117,118]. Neutravidin is water soluble, engineered to be zwitterionic, and neutral, and thus does not cause massive non-specific binding in most biosensor applications. It is, however, known that this coupling strategy significantly reduces circulation times of nanoparticles *in vivo* [75]. A surface fully covered with neutravidin might thus not be expected to be equally nonfouling as the unfunctionalized spacer interface.

The use of biotin-avidin as a coupling strategy is also illustrative from another point of view: how to ensure specific coupling and orientation. Biotinylation of ligands for proteins and peptides is mostly carried out using biotinylation kits; such kits add a biotin to e.g., surface-exposed amine groups on the peptide. Only rarely can it be ensured that there will only be one biotin per ligand with this approach. Biotin might also be coupled to the binding domain through this approach, which leads to reduced specificity and selectivity. A range of orientations are also possible for the immobilized ligand due to the random distributed of biotin sites. Furthermore, each ligand can, depending on the density of biotin groups and surface-bound avidin, bind to more than one site on the surface. This increases the risk for denaturation of the structure and lowers the overall density of recognition elements. It is even possible that every immobilized ligand is rendered inactive. The problems of multiple functional groups for attachment of each ligand can be generalized to other systems than for biotin-avidin. They are also valid for chemical immobilization strategies making use of peptide surface functional groups, such as coupling to amine or carboxyl groups, which are abundant on the recognition element surface.

It is also important to point out that selectivity does not automatically mean high affinity. In fact, biotin-avidin recognition is so often used to demonstrate proof-of-principle for a new biosensor, because it has an unusually high affinity, in the low pM regime, which is seldom found otherwise. Antibodies, which can be highly specific, have affinities in the range of 0.1 to 10 nM. The affinity is often reduced by partial denaturation and sub-optimal orientation at a biosensor interface. A nanoscale sensor aimed to detect low-abundance analytes (sub-nM concentration) therefore has to take into account that a certain sensing site might only be populated by a single analyte molecule for a short time before the molecule desorbs again. Related to this point is that a recognition interaction can indeed be so weak that multivalent binding is required for detection. As discussed in a separate section below, multivalent interactions are common in biological systems; in fact, they serve as motivation to design biosensors which can explore and use such interactions. However, when multivalent binding is required for capture of an analyte, a sufficient number of closely spaced recognition elements in the correct geometrical configuration must be present on the sensing element. A sensor surface is typically two-dimensional, which puts geometrical constraints on the art of multivalency that can be applied. Using flexible spacers for the attachment of recognition elements can help circumventing this constraint.

#### 2.9.2. Binding Fluctuation Analysis

Here it is also in place to mention that nanoscale sensors with low noise and high time resolution can use low affinity recognition elements advantageously by extending the analysis of the acquired data. A very detailed analysis of the affinity and the ligand recognition interaction can be performed if the sensor size and functionalization allows for detecting single or few molecule interactions. An analysis of the fluctuations of the sensor response, which corresponds to fluctuations in single-site occupancy, will reveal information not only on the affinity but also on *k*_on_ and *k*_off_ of the interaction [119] The principle is the same as long established for single ion channel readout used to probe single translocation events of ions or charged molecules [120]. Additionally, it can reveal sub-populations and broad heterogeneities if multiple types of interactions are possible [121].

### 2.10. Summary of Capturing Analytes at the Sensor Element

In summary, a range of well-established recognition elements, mostly recruited from already existing biological molecular systems, exist and have been adapted to surface-based sensor surface modifications. Their proper function as recognition elements is crucially dependent on how they are linked to the surface both in terms of chemistry and geometry. The latter aspect is more crucial when designing surface modifications for nanoscale sensors than for traditional biosensors. So far, most work has been focused on evaluating the applicability of existing surface modifications designed for macroscopic sensors. There is thus significant room for improvement with respect to optimizing the surface anchor, the spacer and possibly also the functional unit for nanoscale sensors. The best strategies for this might differ and require further innovation.

## 3. Alternative Surface Patterning Strategies

As an alternative to the use of material selective (orthogonal) surface modifications, which relies strictly on careful selection of the anchor groups, various printing methods can also be used in order to generate small-scale patterns suitable for different types of biosensor applications. Below, follows a brief description of some of these, focusing on the challenges of generating multifunctional small-scale patterned surfaces.

### 3.1. Lithographic Patterning of Physisorbed Macromolcules

**Figure 9.**
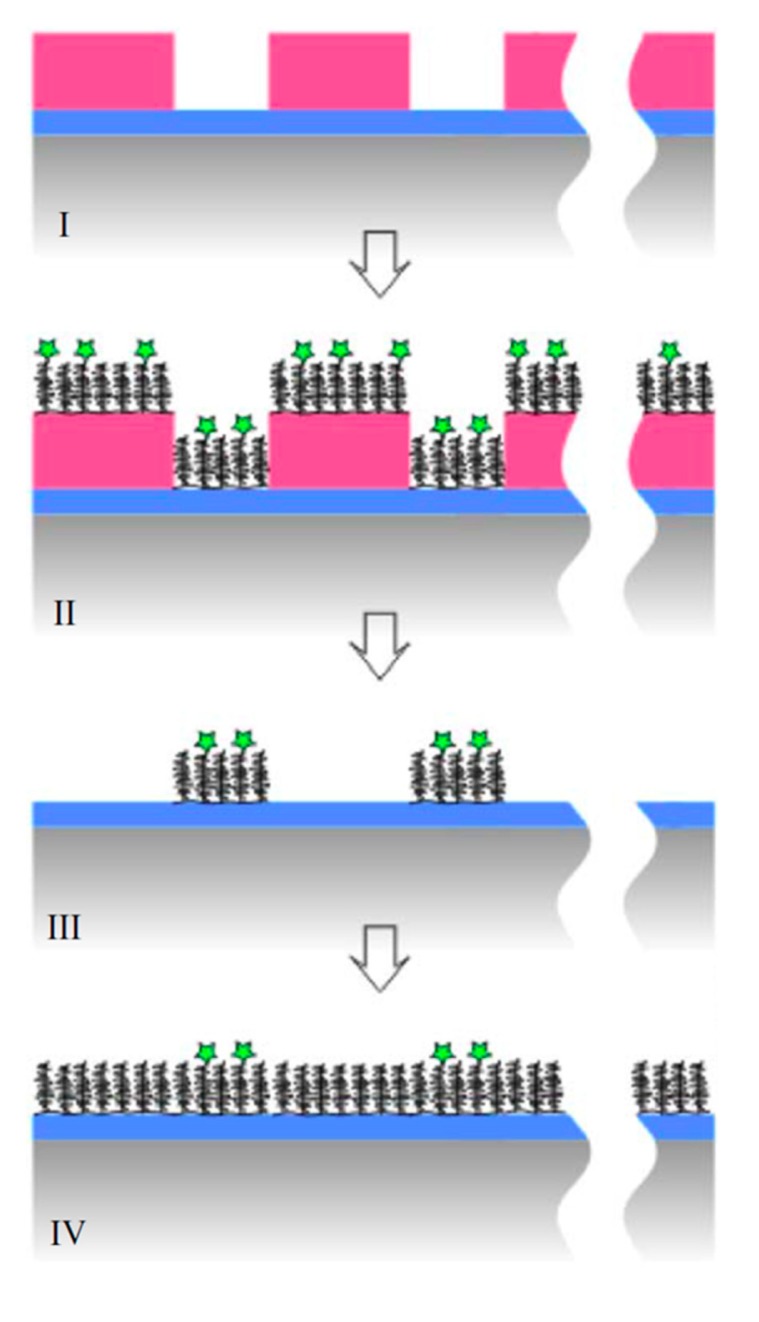
Schematic of the MAPL process, showing how it can be used to convert a photoresist patterned oxide substrate into a surface with well-controlled biointeractive patches in a non-interactive background [122]. The sample composed of a photoresist pattern (stage **I**) is dipped into a solution of functionalized (e.g., biotin or RGD) PLL-*g*-PEG (stage **II**). The functionalized PLL-*g*-PEG adsorbs strongly to the bare oxide surface. Next, the PR is removed with an organic solvent without altering or desorbing the polymer immobilized on the oxide, but lifts off polymer on top of the resist (stage **III**). Finally, the sample is incubated in a solution of nonfunctionalized PLL-*g*-PEG to render the background resistant to the adsorption of biomolecules (stage **IV**).

A patterning concept which has been applied successfully to macromolecules anchored by physisorption is lithographic patterning. This was first described for large areas using photolithographically defined patterns of resist and lift-off, and molecular assembly patterning by lift-off (MAPL, Figure 9) [122]. In this approach areas on the substrate which should first be functionalized with e.g., a functional version of PLL-*g*-PEG are exposed while the remaining surface is covered by developed resist. Assembly of the functionalized PLL-*g*-PEG, e.g., PLL-*g*-PEG-biotin, might occur everywhere, but when the photoresist is subsequently lifted off, the PLL-*g*-PEG-biotin in these areas is lifted off with the resist. The free areas are then exposed to differently functionalized (or typically unfunctionalized) macromolecules like PLL-*g*-PEG. It is advisable to create the functional part of the pattern first and backfill with a purely nonfouling species since any defects in the functional coating caused by the multi-step processing will then be “healed” by adsorption of the nonfunctional molecules in the defects. With the reverse process functional binding sites might instead end up in what should be non-fouling areas of the pattern.

The standard MAPL strategy cannot be applied to nanoscale sensor applications, due to the fact that sufficiently high resolution photolithographic patterning cannot be achieved. However, by combining the MAPL approach with nanoimprint lithography (NIL), patterns with feature sizes of 100 nm have been produced [122] and with extreme ultra-violet interference lithography even down to 50 nm [123]. A polymer-resist-free patterning approach built on the MAPL principle was also developed recently. A popular strategy to produce nanostructures, especially high aspect ratio sensor nanostructures, is to etch substrates with several films of materials deposited on top of each other. Reactive ion etching is preferably performed through metal masks of e.g., chrome, which are inert to the etch gases. It was recently demonstrated that chrome etch masks can be used also as lift-off masks for patterning of PLL-*g*-PEG after the etching step [124]. With this method the mask is left in place after reactive etching of the structure. Then the molecular assembly by physisorption of PLL-*g*-PEG is performed, the chrome mask is removed by a short acid etch which is non-destructive to the polymer, but which removes the polymer on top of the chrome structure. The free, previously masked surface is then backfilled with another macromolecule. Thus, the MAPL concept has in several incarnations been extended down to the true nanoscale necessary for small-scale sensors such as nanoplasmonic or nanopore electrochemical sensors [122,124] However, it is important to keep in mind that if the nanoscale sensor element and its surface functionalization is not created in the same lithographic step, *i.e.*, using only one lithographic mask, then the nanopatterns have to be aligned with nanometer precision. This is beyond current state-of-the-art and severely limits the applicability of any direct lithographic method for surface functionalization of nanoscale structures.

### 3.2. Nanoscale Molecular Surface Modification through Printing

Microcontact printing is a hugely successful method to create molecular patterns on the micron scale. The technique makes use of soft stamps, typically of PDMS, with a topography that is the mirror image of the pattern that should be created on a substrate [125–127]. The stamp is inked with a solution of the molecule that should be deposited onto the pattern and the stamp is then applied with pressure to ensure a conformal contact to the substrate. Molecules on the stamp transfer to the substrate during contact. Both physisorption and chemisorption can occur depending on what molecular attachment strategy is preferred. SAM [126], functional PEG brush [128,129], lipid bilayer [130] and protein [129,131,132] patterns have all been created in this way. Application of contact printing and related methodologies to molecular patterning on nanoscale sensors awaits further developments; we will therefore only briefly describe the challenges that need to be addressed to reach this goal.

First, the demands on the stamp material design increases as patterns have to be created that are smaller than the micron or sub-micron replicas typically used today. The pillars creating the contact as well as the stamp body have to be soft, since conformal contact has to be achieved. However, slender or high aspect ratio nanoscale polymeric structures tend to collapse laterally or bundle due to capillary forces and other surface effects [127], which put limits on their softness relative their aspect ratio and spacing. Similarly, for nanoscale sensor objects that are well spaced (low aspect ratio of the noninked regions) the inverse problem of sagging can occur [127]; this leads to molecular transfer to undesired areas of the sensor substrate.

Second, the molecular transfer has to occur onto already fabricated sensor substrates. This requires alignment of the stamp with a precision of a few nanometers onto the sensor. The alignment step can only be avoided by the use of orthogonally binding anchors as discussed for the orthogonal molecular self-assembly approach, which removes the main advantage of the contact printing method. Nanometer precision in alignment is beyond today's standard methodology with the exception of in combination with direct writing as will be described below. Misalignment will lead to, at the very least, severe edge effects from missing or overlapping functionalization and passivation of the sensor elements. Such defects in the grafted layer will significantly compromise the specificity of the biosensor signal.

Third, a sufficient amount of polymer, protein or other surface modification molecule to self-assemble into the correct density has to be transferred to the surface. The density has to be sufficient to meet all the requirements discussed above for spacer and functional groups. This requires that the transferred molecule has a suitable conformation, e.g., a collapsed for polymers forming polymer brushes. This requires that the molecules are applied in a solvent. Pressing a solvent-containing stamp tends to produce wetting of the printing solution also in the vicinity of the contacting pillar. Naturally, this limits the pattern definition by molecular diffusion in the wetted film [127,133,134]. These effects can be irrelevant on the micron scale, but can be severe when pattern definition is defined on the nanometer scale [134]. An excess of molecules have to be contained in the stamp to achieve complete monolayer density. This implies the use of hydrogels for macromolecules. For example, for stamping PLL-*g*-PEG a very soft polyacrylamide hydrogel stamp had to be used in an inverted stamping scheme instead of PDMS [135]. Pattern resolution is further limited by such highly deformable stamps.

Fourth, many geometries which take advantage of the unique aspects of nanoscale sensors to, e.g., minimize sample consumption or produce directional sensing make use of inherently three-dimensional nanoscale design of the sensor elements. Contact printing on these length scales is an inherently two-dimensional method; thus, it cannot guarantee homogenous distribution of the patterned molecules into e.g., nanopores. The application of contact printing might be limited to planar sensor configurations even if molecular printing on the nanoscale would become routine.

### 3.3. Nanoscale Molecular Surface Modification through Direct Writing

Until recently, molecular patterning on the nanoscale was only practically feasible by either vacuum physical deposition methods. These are not suitable for biological compounds or for the type of molecular self-assembly described above. However, the advent and continuous improvement of dip-pen lithography [136,137] and related methods have opened up the possibility to write molecular patterns with nanometer precision. Using an inked, nanosized tip, in advanced designs connected to a reservoir for continuous writing, and the control principles of scanning probe microscopy, molecules can be deposited by nm precision onto a substrate [136,137]. Massive parallelization making use of thousands, or more, of tips writing on the surface simultaneously has significantly reduced the time for molecular pattern deposition over the large areas typically employed for nanoscale biosensors [138].

Application of direct writing to nanoscale biosensor functionalization has the advantage over e.g., contact printing that the structure can be imaged and individually aligned with the inking tip. Confining a reaction locally has enormous advantages for biochemical pattern creation. Also in the case of direct writing, the goal is to transfer the functionalized spacer molecules or reacting a ligand to a functionalized spacer in the defined area of writing [137]. Therefore, the correct environmental conditions have to be met for those reactions and the surface density of the functionality has to be controlled similarly to what was discussed for self-assembly patterning. This is a bigger challenge when working with small rather than large volumes of solutes. Tip-assisted writing of nanoscale patterns suitable for biosensors has been demonstrated through “nanoshaving”, “nanografting” and grafting from of polymer brushes from deposited or activated initiators [139]. Combined with backfilling of initiators or of polymers adhering to surrounding areas these techniques could be used to functionalize nanoscale sensors without employing orthogonal chemistry. This constitutes a significant simplification despite adding an additional and quasi-serial processing step to the surface modification process.

Inking has in contrast to printing also been applied to 3-D nanoscale structures. A cavity can be filled by using tips which can dispose larger amounts of liquid than a traditional hard-tip dip-pen. By filling a nano- or microcavity, walls of the cavity can be functionalized through localized self-assembly without direct contact with the tip [140]. Versions of dip-pen lithography using soft or hydrogel tips have also recently been demonstrated; such soft tips put larger solute volumes in contact with the surface under conformal contact without losing confinement compared to a hard stamp or tip [137,140,141]. Writing on topographically patterned substrates and substrates with material contrast poses additional challenges in terms of controlling the wetting as the molecular sample is ejected in a solvent. When solvent is ejected, it must wet the substrate surface sufficiently better than the ejecting tip in order to transfer to substrate, but it cannot wet the substrate so well that it spreads over a larger area than what is intended to be patterned. Preferential spreading of the solvent over adjacent surface areas with a different surface chemistry has to be avoided. Generally, molecules for patterning act as detergents in the solvent which promotes wetting. It makes it a complex task to control simultaneously wetting, deposition and chemical reactivity and therefore limits the creation of general protocols for use of dip-pen lithography for any nanoscale sensor configuration.

### 3.4. Multivalency and Mimicking Fluid Biointerfaces

Discussions on biosensor detection often implicitly assume that a surface bound capture agent binds to the analyte with sufficient affinity to allow for the detection of its presence at the sensor element over the measurement time scale. Therefore, surface modification schemes are generally based on recognition units being immobilized at the biosensor interface at a sufficient or optimal density for detection. We have so far discussed this case. However, in nature, most recognition processes are of low affinity. Multivalent interactions to boost the total affinity are more the rule than the exception for important processes [53]. This is especially true for interactions occurring at cell membranes. Fluidity of the recognition elements in the membrane enables ligands to be recognized through lateral diffusion; thus high avidity via multivalent binding can be achieved. The avidity is increased by the possibility to locally accumulate binding sites that diffuse to the interaction zone and bind in equilibrium with the reservoir of binding partners at the membrane interface.

Thus, many recognition phenomena such as transcription factor binding to DNA [142], phosphoinositide-mediated protein binding [143] or carbohydrate-protein binding [144] are the integral of many simultaneously occurring, polyvalent low affinity events occurring in the necessary geometrical and sequential constellation, for which each single binding event is transient. Such events can in the best case only very inefficiently be probed by the conventional surface modification schemes discussed so far.

**Figure 10.**
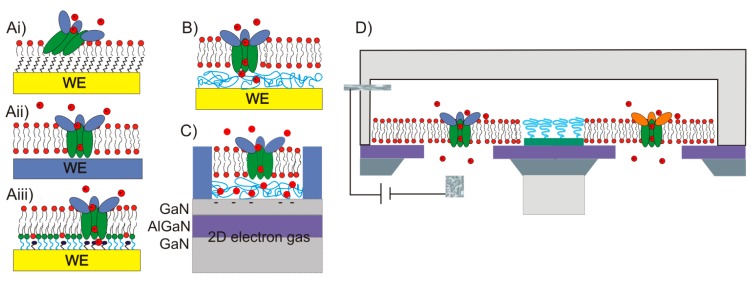
Schematics of different membrane architectures implemented for biosensing redrawn from [148]. (**Ai**) Hybrid bilayer consisting of a self-assembled monolayer and a lipid monolayer which can be self-assembled on moth biosensors; (**Aii**) A solid-supported lipid bilayer suitable for cantilever (acoustic), waveguide and silicon nanowire sensors; (**Aiii**) A tethered supported lipid bilayer with spacers suitable for plasmonic and impedance measurements; (**B**) A hydrogel-cushioned lipid bilayer decoupled from the solid support; (**C**) A miniaturized cushioned lipid bilayer gating a field-effect transistor (so-called BIO-FET); (**D**) Nanopore-spanning lipid bilayer for nanoscale optical and electrochemical sensing.

The by far most common approach to mimic multivalent recognition processes in biosensor interface design is to implement a fluid membrane as the sensor surface modification. The key to success is to achieve the restricted mobility of recognition elements that characterizes such ligands in biological systems. In biological systems this is conferred through the scaffold, e.g., cell membranes, to which the recognition elements are attached. Fluidity of a surface coating implies that it has to consist of a liquid or liquid crystalline system. The most well-known and frequently implemented liquid crystalline surface modification is the lipid bilayer, which is a simplified model of the cell membranes. It has been assembled onto biosensors as well as for other applications in many different incarnations (Figure 10) [145–147]. As a reductionist version of cell membranes, phospholipid molecules self-assemble into surface-adhering membranes with the hydrophilic head oriented towards the liquid environment and the hydrophobic tails facing each other by a variety of methods [145,146].

By a popular method, lipid vesicles of the desired composition are first spontaneously formed and homogenized to a diameter in the 100 nm range in solution; thereafter they are adsorbed onto the solid surface of the biosensor. The liposomes fuse together into a planar supported lipid bilayer under suitable conditions of pH, temperature and counter-ions for the combination of lipid composition and surface material [65,149–153]. This method is popular, but it has mainly been demonstrated to yield high quality surface supported membranes on a limited set of oxide surfaces, e.g., SiO_2_, TiO_2_ and ITO. Environmental conditions and additives that promote fusion to a planar membrane have been introduced [150,151]. The most noteworthy is the use of viral fusion peptides to form supported lipid membranes by liposome self-assembly also on substrates where they spontaneously do not form, such as on Au [154]. The liposome fusion method has been complemented with other surface modifications such as tethered lipids in a self-assembled monolayer to promote fusion also to metal surfaces [155–158]. It has also been applied to nanoscale porous surfaces for integrated sensing [148,159,160]. A supported lipid membrane conforms to surface features larger than ca. 20 nm or smaller than a few nm [161]. It retains fluidity similar to a biological membrane [146]. This can only be achieved when the individual lipid-surface interaction is very weak, but the internal cohesion of the membrane is very strong due to the hydrophobic core. The membrane can thus as a whole attach strongly through the sum of a very large number of weak attractive interactions. By design of nature, commonly used phospholipid membranes can be regarded as non-fouling to most biological fluids free from lipid-binding molecules or specifically lipid degrading lipases, due to a combination of stable hydration of the headgroup region, predominantly zwitterionic charge of the headgroups, the low number of defects, the ability to self-heal and the repulsive entropic contributions from membrane fluctuations and fluidity [162–164]. Supported lipid bilayers have already been implemented for numerous sensing applications in the laboratory setting [146–148]. However, the lack of commercial examples indicates that the mechanical fragility poses severe problems for field applications. Implementation of lipid membranes to functionalize biosensors is also, but rarely, used to probe interactions with membrane bound species or for sensing of multivalent binding. However, that lipid membranes indeed can be used to investigate multivalent binding has also recently been inferred from binding events taking place to lipid membrane functionalized biosensors [143,165–168].

Assembly of planar lipid bilayers by liposome fusion and other means is restricted on the nanoscale due to the very high energy associated with exposing edges of a membrane. An energy gain from opening up of a liposome to exposing edges thus has to be compensated by greater reduction in energy from the increased surface adhesion and can still comprise a kinetic barrier to rupture [169,170]. Supported membrane formation is thermodynamically rapidly increasingly favored with increasing supported membrane area. Thus, higher adhesion energy is required to form a smaller membrane, which strongly will affect membrane properties such as fluidity. The edges of lipid membranes are also by nature more hydrophobic and therefore pinning points for large membrane molecules and sites of high binding energy for molecules in solution. Thus, one risks having an ill-defined surface modification at the edges of the sensing areas where nanoscale biosensors are often most sensitive. Furthermore, the nanoscale sensor area has to incorporate a sufficient number of tethered recognition elements to perform multivalent binding. An obvious way to circumvent the nanoscale restriction is to expand the allowable area of lipid surface modification to a greater surface than the sensor element [26,171]. When the recognition elements are mobile, recognition can in principle occur anywhere on the surface by multiple ligands and be transported by diffusion within the membrane to the sensing site. This requires homogenization of the surface chemistry, e.g., by covering the entire surface with an oxide film suitable for lipid membrane assembly. A major drawback with this approach is that a major advantage of nanoscale sensors is not utilized. The functionalization of the recognition element is not patterned and therefore identical to that of a macroscale sensor. Homogenous surface modifications additionally tend to reduce the sensitivity of nanoscale transducers because the biorecognition reactions are forced to occur at an appreciable distance from the sensor surface.

Given the severe practical obstacles to implement supported lipid membranes as surface modifications on the nanoscale, other membrane geometries should be considered. Lipid membranes of complex composition, including peptides, glycolipids and other suitable recognition elements, are most easily assembled in lipid vesicles. The composition and size of lipid vesicles can be controlled in a relatively straightforward manner. At a size of 100 nm in diameter, a liposome has roughly the size of the sensing element of most nanoscale sensors. Direct assembly of liposomes by a tether to a nanoscale sensor that has first been made non-fouling by adsorption of a spacer layer and patterned to capture the liposomes allows placing a stable, functional lipid membrane recognition element patterned exclusively on the sensor elements [37,38]. However, potential drawbacks to consider are the highly curved membrane geometry of small vesicles, the often restricted space in the most sensitive tethering region and that many nanoscale sensors, e.g., nanoplamonic sensors, have a shallow penetration depth; they are often insensitive to recognition events occurring at the far side of the liposome [37].

## 4. Conclusions

In summary, many different surface functionalization strategies exist which in principle are compatible with small-scale sensors. However, their application to nanoscale sensors requires careful consideration of the geometrical constraints that are imposed. The optimal design depends critically on the specific application. In general, modular approaches to functionalization that exploit self-assembly of the molecular coatings specifically tailored to the materials contrast offered by nano- and microscale sensors are advantageous; this further allows rapid retailoring of the molecular structure to meet the demands of new application areas. However, the fact that the size of nanosacle sensors is similar to the size of the molecular building blocks to functionalize them makes precise control over the spatial distribution of the molecular binding events essential. Further development of the different patterning approaches, including improvement in selectively binding anchor chemistry, nanolithography, immobilization methods for recognition elements and approaches to address also multivalent recognition events are being addressed by a large community. Improved nanofabrication schemes are likely to reduce the uncertainty imposed by ill-defined material compositions at the boundary between different materials.

An interesting approach is to use the actual transducer elements as active elements to aid the selective patterning of these areas. For instance, surface potential, temperature, magnetic fields *etc*. can locally be manipulated to drive selective functionalization on the sensor element [172,173]. In a variation on that principle, the lateral mobility of supported lipid bilayers can be used not only to aid multivalent interactions, but also to position probe elements by exerting external electrical [174–176] of hydrodynamic [177] forces to concentrate membrane bound components at surface-based sensor elements.

The future development in this line of research relies heavily on further development and understanding of surface functionalization strategies that allow binding of target molecules only to the sensitive regions. The surrounding regions should be kept inert and highly specific and selective recognition elements should be patterned only on the sensitive areas. Such functionalization can concentrate the adsorbing analyte molecules on small areas with high transducer efficiency [85,178]. Proteins reaching the inert regions are repelled, allowing them to diffuse or be driven by convection to the bioactive areas where they correspondingly bind at a considerably higher rate than if binding occurs over the entire surface. Given that the sensitivity in terms of average surface coverage is similar for nanoscale and macroscopic sensors, this provides a real advantage of nanoscale sensors compared to conventional techniques such as QCM, SPR and EI, where the entire surface is sensitive. Despite the enormous work on nanoscale sensors and the tremendous attention they have attracted in the scientific community over the last 10 years, one can say that the design of optimal surface functionalization protocols to exploit their potential remains at an early stage of development.

## Figures and Tables

**Table 1.** Compatibility of weak and strong anchors to different surface materials.

**Type of Anchor**	**Graphic Example**	**Anchor Examples**	**Surface Materials**
**weak anchors (physisorption)**	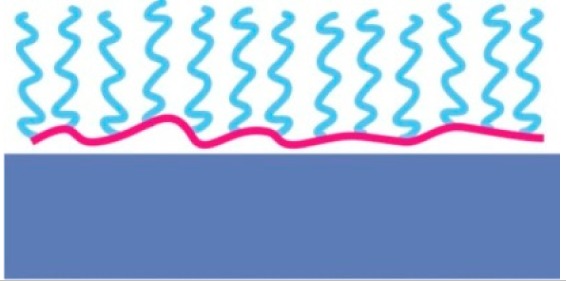	amino acids; proteins polyelectrolytes hydrophobic chains hydrophilic lipid head group	flexible but non-specific metal oxides; charged surfaces hydrophobic surfaces hydrophilic surfaces
**strong anchors (chemisorption)**	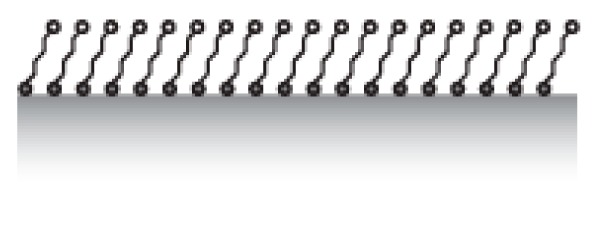	Silanes	SiO_2_ (metal and non-metal oxides/hydroxides)
		Thiols	Au, Ag, Cu, Pt, ITO
		phosph(on)ates	TiO_2_, Al_2_O_3_, TiO_2_ and many other transition metal oxides
		catechols	TiO_2_, Fe_3_O_4_ (metal oxides)

**Table 2.** Combinations of anchors and spacers that render substrates inert to non-specific biomolecular binding.

**Type**	**Graphic Example**	**Advantages**	**Disadvantages**	**Surface Materials**	**Examples**
**Proteins**	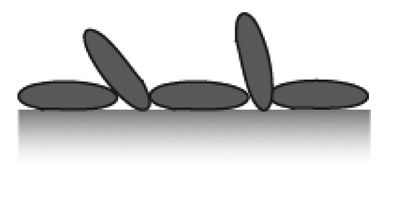	easy to handle general biocompatible	unspecific to surface material, surface patches exposed, non-specific binding and orientation	relatively general	bovine serum albumin fibrinogen milk proteins
**Self-assembled monolayers**	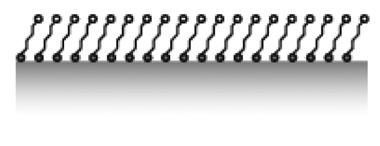	applicable to many surface materials receptor exposure good	small substrate defects alter function, low resistance to fouling	Au, Ag, SiO_2_, TiO_2_, Al_2_O_3_	OEG-thiols OEG-silanes
**Surface-grafted polymer brushes**	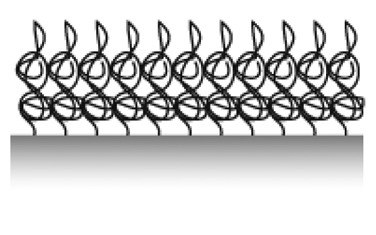	surface chemistry specific grafting grafting from of any spacer mix and match functionality by grafting to	difficult to control surface density, low availability of functional group	Au, Ag, SiO_2_, TiO_2_, Al_2_O_3_, Fe_3_O_4_, Fe_2_O_3_	PEG-thiols PEG-silanes PEG-DOPA_3_ PEG-nitrocatechols
**Physisorbed graft copolymer brushes**	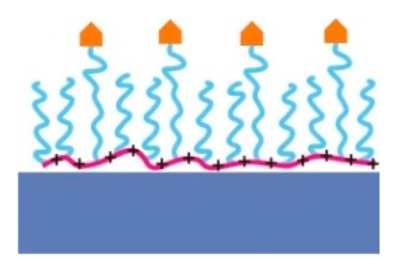	applicable to many surface materials low defect intensity known density of functional groups	large surface area per macromolecule, low availability of functional group, non-specific surface attachment	Au, Ag, SiO_2_, TiO_2_, Nb_2_O_5_, Si_3_N_4_, Al_2_O_3_,	PLL-*g*-PEG PLL-*g*-PMOXA Pluronics^®^
**Amphiphilic membranes**	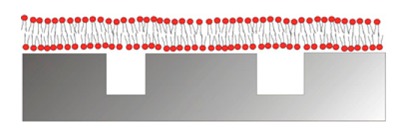	cell membrane mimic multivalancy through lateral mobility	cannot be dried, limited to certain surface materials	SiO_2_, TiO_2_, ITO, hydrophobic surfaces	Lipids block-co-polymers
